# Active Thermography in Diagnostics of Timber Elements Covered with Polychrome

**DOI:** 10.3390/ma14051134

**Published:** 2021-02-28

**Authors:** Milena Kucharska, Justyna Jaskowska-Lemańska

**Affiliations:** Department of Geomechanics, Civil Engineering and Geotechnics, AGH University of Science and Technology, Al. Mickiewicza 30, 30-059 Cracow, Poland; kucharska@agh.edu.pl

**Keywords:** active thermography, non-destructive tests (NDTs), semi-destructive tests (SDTs), timber, knot area ratio, polychrome

## Abstract

The contribution of natural wood defects such as knots is an important factor influencing the strength characteristics of structural timber. This paper discusses the use of active thermography in the timber diagnostics, particularly in the determination of the knot area ratio (KAR) in elements covered with paint coatings. Moreover, on the basis of thermal images, the localization for the subsequent semi-destructive tests (SDTs) was established. Three different sources of external energy supply were used in the studies: laboratory dryer, air heater and halogen lamps. The active thermography tests were performed on elements made of three wood species (fir, pine and spruce). The specimens were covered with varying layers of paint coatings and primers, to reflect the actual condition of the historic structural elements. The obtained thermal images enabled the estimation of the KAR, due to the difference in temperature between solid wood and knots occurring therein. It should be noted that the results were affected by an external energy source and subjective judgement of the operator. Moreover, active thermography could be an effective method for the indication of the regions within which SDTs should be performed in order to properly assess the technical state of an element covered with polychrome.

## 1. Introduction

In optimal conditions, timber maintains its extraordinary durability; however, in conditions of regular exploitation of buildings, it deteriorates more rapidly than other building materials. Therefore, integrated elements, especially in historic buildings, more frequently require repairs and strengthening than those made of other materials. Furthermore, timber construction is considered to be a sustainable building style, due to timber’s being a renewable resource and the little requirement of production [1,2,3]. In order to construct timber elements properly, it is necessary to determine their actual technical condition, i.e., their physical and mechanical properties [4].

Testing of timber structural elements, due to their impact on the material structure, can be divided into destructive, non-destructive and semi-destructive tests. Destructive testing in historic buildings is generally prohibited from the preservation point of view; hence, it is usually entirely impossible to apply. For this reason, the standards and normative guidelines primarily recommend the use of non-destructive test (NDT) and semi-destructive test (SDT) methods [5,6,7]. Non-destructive testing, which includes visual inspection, pre-load tests and tests based on ultrasonic echo techniques, does not generally provide sufficient information on the mechanical characteristics of the tested element [8,9]. The application of semi-destructive tests (e.g., sclerometric or drilling resistance tests) provides a qualitative assessment of the material, yet requires careful selection of the testing location in order to obtain results representative for a given material type [10]. The location of such tests should be devoid of the influence of any natural wood defects, such as knots or cracks [11]. In most cases, it is necessary to apply various testing methods to determine the technical condition of the structure.

**Figure 1:**
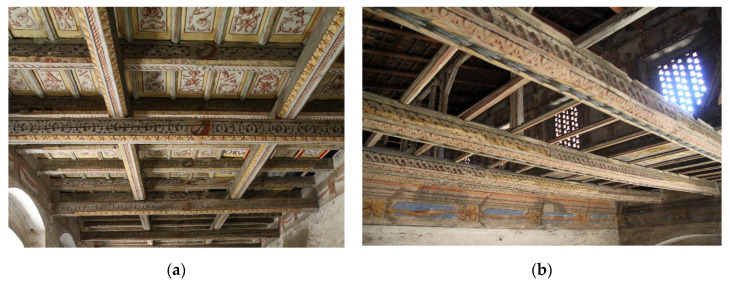
Examples of polychrome ceilings requiring technical condition analysis in Gorzanów Palace, 2015: (**a**) ceiling in a lounge chamber, (**b**) ceiling in a theatre chamber.

The problem of determining the existing load-bearing capacity of timber structural elements, usually ceilings and roof frames, appears during each restoration or modernization of historical buildings and structures [12]. It should be taken into account that, in such objects, the natural structure of the wood, which enables the proper selection of the location for SDTs or the determination of the knot area index (KAR), is often covered. In particular, this concerns historical polychrome elements, which are of substantial cultural heritage. Figure 1 shows two examples of historical Renaissance wooden ceilings of the Gorzanów Palace, which, before further conservation and subsequent exploitation, required an evaluation of their technical condition, to confirm their satisfactory quality and thus their safety.

Polychromes in timber historic buildings are multicolored decorations of walls and vaults inside the facilities. Since the Renaissance, they have been the most frequently used method of decorating interiors of both secular and sacred objects. In many cases, paint coatings conceal the actual texture of the material. Therefore, it becomes difficult to determine the defects and structural characteristics of wood required to adopt the timber quality class. A useful instrument for the detection of damage and natural defects in wood hidden under polychrome is thermography, which reveals temperature differences of the material also underneath the external surface.

The idea of thermographic tests is focused on temperature registration of an object and its imaging within the medium infrared spectrum. The thermographic examination consists in measuring the intensity of infrared emission of the object, which is proportional to its temperature [13]. There are two types of infrared thermography: passive, which is widely used in practice, and active, which is nowadays not very widespread in the buildings structure examinations [14]. However, in the case of other structures, such as composites and metals for aerospace structures, the active thermography is widely applied [15]. Passive thermography uses natural radiation of the examined object as a source of heat, while the active is based on the registration of reactions to external and independent thermal stimulation [16]. Therefore, both approaches differ from each other by the implementation of an external energy source. In order for passive thermography to be considered valid, the tests must be performed in suitable hygrothermal and weather conditions. In active thermography, the following systems are usually used as an external energy source: various types of lamps, air heaters, ultrasonic heads, eddy current generators and infrared illuminators [13]. Each of the external energy sources needed in order to produce a thermal contrast between the sound material and a surface or subsurface of defect uses different mechanisms: conduction, convection, thermal radiation, acoustic or ultrasonic wave propagation, electromagnetic radiation, mechanical stimulation, electrical or chemical mechanism. Thermal images obtained with optically stimulated thermography are prone to various noise, including external reflections, variations in optical properties of a sample and non-uniform heating resulting from the selection of the external energy source. In order to reduce the influence of these imperfections on the image results, signal-processing techniques, such as differential absolute contrast, thermographic signal reconstruction, correlation extraction algorithm and many more, are applied [15]. General guidelines and various methods for the application of active thermography were presented in References [15,16,17,18,19], among others.

In civil engineering, thermography is used for the non-destructive diagnostics of building structures, most often for validation of quality and integrity of building envelopes [20], in energy audits of buildings [21] and diagnostics of building materials, including wood [13,22,23,24,25,26]. It should be noted that active thermography is successfully used in the diagnosis of isotropic materials. Its application in the examination of wood, which is inherently anisotropic material, is complicated. This is primarily related to changes in density both within successive annual growth rings and its irregularities in the area of natural defects, among others knots, cross grains and reactive wood, as well as the hygroscopic properties of wood.

In the field of timber investigations, thermography has been applied to determine the technical condition of wood and growing trees, especially through detection of deteriorations and voids that compromise its structure, stability and durability [27]. In the literature, the most commonly discussed is the implementation of thermal imaging to determine the density of timber [23,28], the location of defects on prepared samples [29] or historical samples [30,31,32] and the determination of moisture using such technique [33]. A separate issue presented in the literature is the determination of emissivity for different wood species [34].

As it was mentioned in the previous paragraphs, thermography provides the possibility of conducting research in historical objects without disturbing their structure. Active thermography can be particularly beneficial for elements covered with different paint coatings, referred to as polychromes, since passive thermography is insufficient to detect differences in wood structure when assuming similar moisture content within the wood [28]. In general, the moisture difference visible in passive thermography images is related to the excessive moisture in fragments of the elements, e.g., the support zones of beams. In the literature, there are examples of its application both for determining the location of natural defects of wood such as cracks or knots under the primer coating [30,31] and for the detection of defects within the paint coating [35]. As it is known, polychromes, as a part of heritage, should be particularly protected. The environment in which polychrome is displayed has a significant impact on their condition and long-term preservation. Hazardous factors to which the objects may be exposed during thermographic testing are mainly excessive lighting and increased temperature. Therefore, the energy source used should be adapted to the sensitivity of the coating [36].

The amount of light damage is determined by the intensity and type of light, as well as the time of exposure. Exposure to average daylight levels of 30,000 lux will cause observable fading of highly sensitive paint in as little as a day to two weeks and of medium-sensitivity paints between two weeks and a year. In the case of museum collections, it is recommended to limit the duration of continuous exposure to light with an intensity of 50–150 lux to three to four months [37]. Bookending the visible light spectrum is ultraviolet (UV) and infrared (IR) radiation. UV radiation will yellow and weaken materials, and IR will cause the surface of objects to heat up [38]. Highly sensitive paints will exhibit UV damage from exposure to unfiltered daylight within several months. It is strongly recommended that UV levels should range within 0–10 microwatts/lumen and definitely not exceed 75 (µW/lm) [39]. The temperature of paint coatings should not exceed 45 °C. In spite of the negative effects of elevated temperatures, the effects of significantly reduced temperatures are more destructive. They make paints more vulnerable to shocks and blows [40].

There are a number of different lamps available for overall or focused and directional lighting use. The UV emitted by quartz-halogen lamps is only moderate, making it substantially lower than that from daylight through window glass and, in general, less than from fluorescent lamps. For short-term exposures, the impact is not significant. Thus, to a limited extent, they can be used in illumination and active thermography research.

In this study, the issue of a knot area ratio (KAR) determination for elements made of coniferous wood, which is most commonly used in Europe, covered with layers of paint coatings without damaging them, was undertaken. KAR is a parameter that indicates the proportion of knots in structural timber. The principles of KAR evaluation are given in Section 2.2.1. The application of active thermography is proposed as a tool for this purpose. Moreover, it was proposed that the results of active thermography tests are to be the basis for determining the location of crucial areas to perform semi-destructive tests. The comparison of the semi-destructive and thermographic tests was conducted.

## 2. Materials and Methods

### 2.1. Materials and Test Elements

The tests were conducted on the test elements made of structural timber. Materials selected for the purpose of the research were the most frequently used wood species in civil engineering structures, i.e., pine (*Pinus sylvestris*), spruce (*Picea abies*) and fir (*Abies alba*). The cuboidal specimens with approximate dimensions of 17.5 cm × 17.5 cm × 35 cm were prepared. Particular wood species (fir—F, pine—P and spruce—S) and side surfaces of cuboids (A–D) were marked on the cross-sectional area, assuming consistent symbols for the entire scope of research. The timber pieces were artificially prepared to match the historic appearance of the wood. The samples were sanded before testing.

In order to reproduce the various surface preparations that occur in historic buildings, three types of primer characteristic for these objects were selected. The chosen surfaces of the elements were covered with glue–chalk plaster or construction putty; the remaining surfaces were left without a primer, which is also often observed in historic buildings. Three basic traditional painting techniques were used in the research, such as pigment combined with protein binder, acrylic and oil paints. Moreover, modern techniques used in antique preservation, such as hydroxypropyl cellulose (Klucel) and alkyd paints, were applied.

Klucel is synthetic cellulose that is used in preservation, e.g., for bonding powdered paint coatings or as a pigment binder for replenishing paint coatings [41]. Klucel M 3.5% was used in the research, and it was combined with natural pigments. Phthalic (alkyd) paints are produced on the basis of alkyd resins and solvents, in which pigment particles are suspended. They are increasingly common in historic buildings, as a form of protection of the texture of timber elements. The egg yolk was used as a traditional protein binder to be combined with pigments [42]. Such an ingredient could be adopted due to content of phosphoprotein (vitelline), which has the ability to combine with water. The yolk was diluted with apple cider vinegar and combined with drying oil. The prepared emulsion was mixed with natural pigments, to form a paint.

Before proceeding with the recreation of traditional paint coating techniques on a wooden surface, the timber was sized with 3% rabbit-skin glue to create a natural protective coating on the surface and reduce water penetration into the wood structure. Rabbit-skin glue is an animal glue created by prolonged boiling of animal connective tissue. It was used both as an ingredient of traditional gesso or paint and sizing on canvas [43]. Afterwards, selected primers were applied to the respective surfaces, and after drying, the elements were covered with the aforementioned coatings.

**Figure 2:**
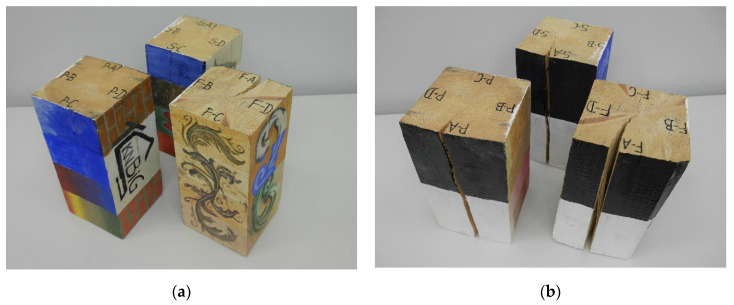
Test element with applied paints: (**a**) front view and (**b**) back view.

Figure 2 presents the test specimens with applied paint coatings with a visible designation of side surfaces. Table 1 summarizes the primers and layers of paint coatings applied on the respective side surfaces. Such an arrangement was maintained for all wood species used in the research program.

### 2.2. Test Plan

The research was divided into three main phases. The first phase included the macroscopic examination of the elements, in which the dimensions of the elements were measured and the total knot area ratio and the margin knot area ratio were determined. In the second phase, the thermographic examination of the elements covered with paint-coatings was carried out, and on their basis, the total KAR and margin KAR were recalculated. The three different sources of external energy for active thermography tests were proposed. Therefore, the second phase consisted of three stages: heating the timber in a laboratory dryer, with an air heater and with halogen lamps. There was an appropriate time interval between tests for wood to regain its initial temperature. Subsequently, the semi-destructive tests were performed on selected surfaces of tested elements. Two methods of SDT were used, namely the sclerometric method, using rebound hammer, and drilling resistance, using a resistograph device.

#### 2.2.1. KAR Determination

One of the factors determining the visual grading strength class of timber is the presence of knots in it, which mainly affect its strength characteristics. According to the standards [44,45], the analysis of the contribution of knots in the element is carried out by determining the knot area ratio for the section with the greatest concentration of these defects, regardless of its distance from the front surface of the specimen. Knots with a diameter of less than 5 mm can be omitted from the calculation.

**Figure 3:**
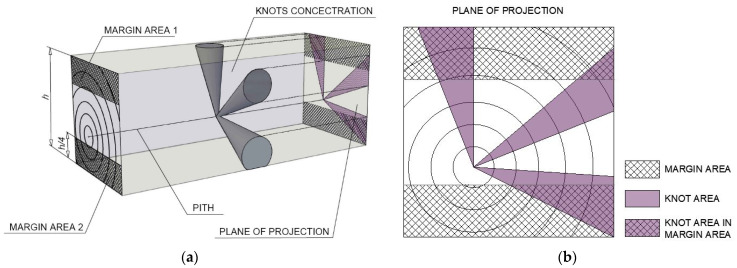
The principles of projecting knots with a division of marginal zones and determination of “worse margin”, based on Reference [44]: (**a**) a spatial view and (**b**) details of the plane of projection.

In order to classify the timber according to standards [44,45], a total knot area ratio (TKAR) is determined, which compares the knots projected cross-sectional area to the entire cross-section of the element, and a margin knot area ratio (MKAR), which compares the knot projected cross-sectional area within the margin zone to the total cross-sectional area of the element. The margin zone is the edge zone occurring along the whole length of each side of the timber, and the MKAR calculations refer to one of two margin zones of the so-called “worse margin” (Figure 3). German standards [46] define KAR as a ratio of the largest knot diameter to the dimension of the graded specimen—thickness or width, depending on the position of the knot [47].

In order to correctly determine the KAR in accordance with the standard [44], it is first necessary to define on the projection plane the diameter and position of each knot that occurs in the critical section. The points determining knot diameters are connected with the pith by straight lines. Depending on the location of the pith—within, on the edge and outside the cross-section—the contour of the knots will be different in the plane of projection. Figure 3 shows the principles of knot projection in the piece of sawn timber with the pith located inside the cross-section. The grading strength class based on, among other thing, the KAR is determined according to the standard [44].

The KAR and MKAR are calculated by using the following equations:

(1) $$\mathrm{KAR}=\frac{K_{\mathrm{area}}}{P_{\mathrm{area}}}$$

(2) $$\mathrm{MKAR}=\max\left(\frac{{\mathrm{MK}}_{\mathrm{area},1}}{M_{\mathrm{area},1}},\frac{{\mathrm{MK}}_{\mathrm{area},2}}{M_{\mathrm{area},2}}\right)$$

where $K_{\mathrm{area}}$—knot area, $P_{\mathrm{area}}$—plane of projection area, ${\mathrm{MK}}_{\mathrm{area},i}$—knot area within the margin zone and $M_{\mathrm{area},i}$—area of margin zone.

#### 2.2.2. Active Thermography

**Figure 4:**
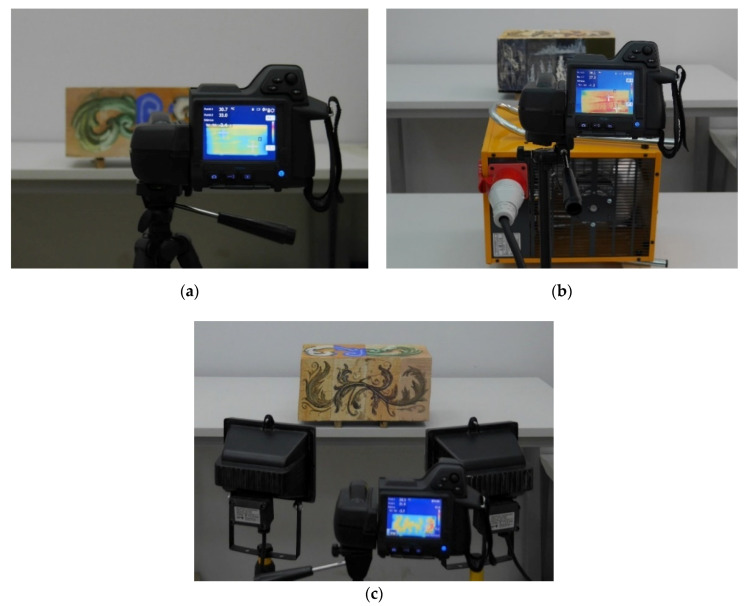
A view of the test stands for active thermography examinations: (**a**) after preheating in laboratory dryer, (**b**) during air-heating and (**c**) during heating with halogen lamps.

Three methods of enhancing the contrast between the surface of solid wood and the surface of knots were used in the study. It was decided to use two different energy supply mechanisms: convection and thermal radiation. For the convection mechanism, warm air from the laboratory dryer (uniform heating of the sample in the whole volume—free convection) and an air heater (heating of the given sample surface, forced convection) were used, whereas for thermal radiation halogen lamps were chosen (surface heating, sinusoidal thermal wave) [48]. The overviews of the test stand for each method were given in Figure 4. For the purpose of this study, a 5 kW air heater and two 400 W halogen lamps were used. The selection of air heater and halogen lamps as energy sources was dictated by their applicability in building objects in an accessible and non-invasive manner. On the other hand, a laboratory dryer was used as a supplementary method, enabling qualitative comparison with the literature data.

An FLIR thermal imaging camera with thermal sensitivity of <0.045 °C at 30 °C, a frame rate of 60 Hz, a spectral range of 7.5 to 13 µm and accuracy of ±2% or ±2 °C was selected for the testing process. The detector is a focal plane array (FPA) uncooled microbolometer with IR resolution of 320 × 240. The focus is both manual and automatic and has a field of vision of 25° × 19° and a minimum focusing distance of 0.4 m. The emissivity ε = 0.95 was adopted for the tests. Convective heating with hot air is uncomplex and emissivity-independent [18]. For tests with halogen lamps, the emissivity may be more influential on the measurement results due to reflected radiation during heating [18]. The emissivity was intentionally selected as the average literature value for different types of paint coatings [49] and timber substrates [34]. The camera was placed at a distance of 1 m from the tested element surface. The parameters were determined experimentally in order to obtain the most visible temperature variation in the surface area in the whole range of the test. The setup parameters of the camera were consistent throughout the entire test series.

In the first stage of phase 2, the components were preheated in a laboratory dryer at 110 °C for 180 s. The cross-sections of the sample were protected with a polystyrene cover, to eliminate heat transfer in this direction. The first thermal imaging was taken after a period of 30 s since the end of the heating process. Within 5 min, a series of 10 photos were taken at 30 s intervals. Such a duration of the test allowed us to record diverse temperature variations of solid wood and knots. The second and third stages were designed to detect temperature variations in the timber during continuous heating. The energy source, both the air heater and halogen lamps, was located at a distance of 60 cm from the specimen surface. The thermal images were taken at 15 s intervals, during 4 min of heating. The application of halogen lamps in such an arrangement generated a light intensity on the surface of the specimen of approximately 24,000 lux. Exposure of a polychrome piece to such lighting would be safe for approximately 15 h of continuous exposure; after that time, fading of the pigments would be perceptible [39].

The temperature difference analysis was performed on each surface for each method with two control points. One of them was each time located within a chosen knot (fixed for all methods); the other one was located on the surface of solid wood (macroscopically assessed as free from defects).

The thermal images obtained during the thermographic examination were analyzed in the AutoCAD computer program. They were rescaled, to match actual dimensions of the specimen, and then the size and position of knots were graphically determined.

#### 2.2.3. Semi-Destructive Tests

**Figure 5:**
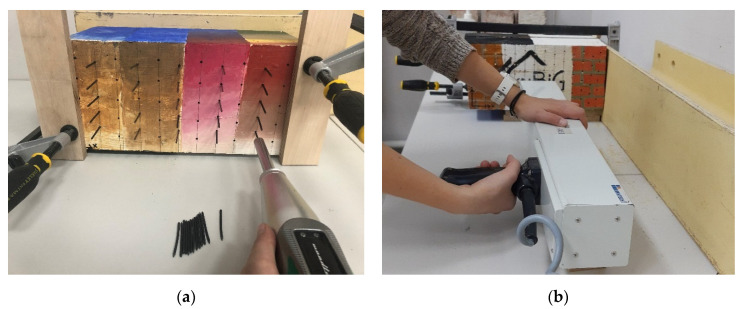
A view of the test stand for semi-destructive methods: (**a**) sclerometric and (**b**) resistographic.

Sclerometric tests were performed by using the Woodtester device of Novatest manufacturer with an impact energy of 2.4 J equipped with steel needles with a hardness of 60 HR, a diameter of 2.5 mm with a conical tip and length of 50 mm and a dial indicator enabling measurement with an accuracy of 0.01 mm. The measurements were taken for the horizontal arrangement of the tool. The value measured was the remaining protruding part of the needle, the difference between the length of the needle and the measured value was treated as the result of the measurement. Analysis involved the needle’s penetration depth (PD) after being double impacted with a woodtester. The tests were performed for a pine sample on surface B. A measuring grid of approx. 2.5 cm × 3.0 cm was adopted. In order to prevent excessive damage and cracking of the wood surface, the tests were carried out in two stages. In the first stage, the examination was carried out on the points located in odd columns and the second stage in even columns. Overall, 68 points were determined. The device used and the specimen during the test are shown in Figure 5a.

Drilling resistance tests were performed with the RinnTech Resistograph 4453-S, equipped with a 1.5 mm diameter drill with a cutting edge with a 3.0 mm–wide top. The device had a drilling rate of 40 cm/min and a resolution of 1/100 mm, dedicated for testing wood with an average density. The torque required to maintain a constant drilling rate corresponds to the resistance of the wood and is registered and plotted in relation to the drilling depth. The measurement results were calculated as the quotient of the area under the drilling resistance diagram and the drilling depth (RM) [50], as a non-dimensional value depending on the device in use. For the pine sample on the surface D, 98 test points were defined in an approximately 2.5 cm × 2.5 cm grid. Measurements of the drilling resistance were made up to a depth of 5 cm from the sample surface. The device used and the specimen during the test are shown in Figure 5b.

The tests in this study deviate from the standard measuring procedure in quantitative terms. In the engineering practice, 9 sclerometric measurements are performed for one region over a surface area of 9.0 cm × 9.0 cm and/or 1 or 2 measurements of drilling resistance. As an example, in the case of a ceiling beam, usually 3 or 4 areas are selected, located within the support zones and in the middle of the span of the element. An extended range of the test area and a large number of measurements were assumed here, in order to map the entire surface area.

## 3. Results

### 3.1. Active Thermography

**Figure 6:**
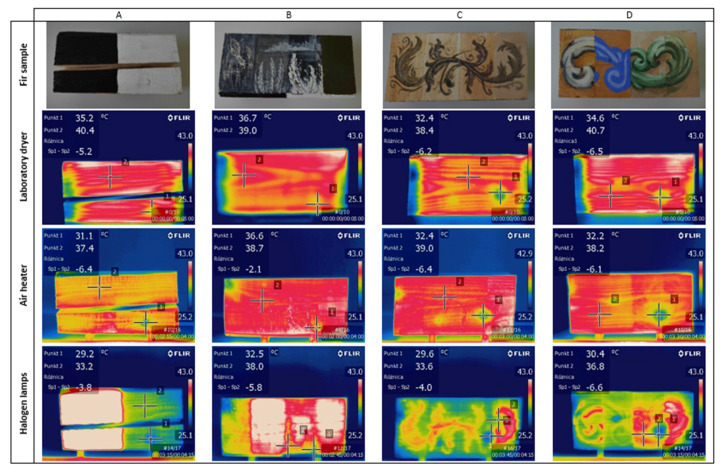
The comparison of the painted surface areas of fir wood with the results of IR thermography: A ÷ D surfaces of tested element.

Figure 6 compares the thermal images for the fir sample subjected to each heating method. Likewise, similar photos and comparisons were made for the other test samples. Thermal images represent the texture of the elements’ surfaces and the defects that occur on them. The location of defects on the thermal images is approximate to reality. It is possible due to the slower acceleration of knots heating, which is connected with their higher density than the density of sound wood.

In the case of wood covered with paint coatings heated in the laboratory dryer, the temperature difference between solid wood and knots was, on average, 5 °C. However, the thermal images demonstrated minor variations in different types of paint coatings.

In the case of the external energy provided with the air heater, after heating for 4 min, the temperature difference between solid wood and knots was approximately 4.7 °C. In this method, the results were significantly influenced by the type of paint, due to its different heating time. Surfaces covered with acrylic and oil paint were the ones to get heated most quickly, while those covered with Klucel-based paint coatings were the slowest. This behavior could be related to the density of paints and, consequently, the thickness of coatings and moreover their thermal properties. In general, the paints with lower density formed a thinner layer, which is more combined with the substrate and their heating temperature is similar to that of wood without polychrome. Timber covered with higher density paints would not warm up as rapidly as the paint coating applied over it. However, as it became more heated, these differences slowly disappeared; thus, such behavior did not significantly affect the final temperature measurement results. It should be noted, though, that after 4 min of heating, the borderline between paints is still visible in the thermal images.

In the case of using halogen lamps, the temperature difference between solid wood and the knots were approximately 5.1 °C after 4 min of exposure. Verification tests were carried out on the analyzed samples, and it was concluded that the modification of emissivity in the range of 0.7–0.95 did not considerably affect the difference in temperature between knot and sound wood, while the absolute temperatures were disturbed. The indications in the thermal images of significantly high temperatures were caused by a measurement error related to the black color of alkyd paint underneath which the knot on the surface A was located. The results of the tests differed with regard to the type of paint coating and its color, which is a consequence of different emissivity. Nevertheless, regardless of the type of paint, dark and warm tones were distinguished much sooner in thermographic images. Exposure of the surface to the light of halogen lamps caused the paint to flash, especially in the case of oil and alkyd paints. This affected their reception by the thermal camera. After preliminary testing using control points equivalent to the other methods, the error was countered by determining the control points on solid wood within a similar color and on the same type of paint coating that the knot was covered with. This allowed us to verify the difference in temperature of the wood and the defect under the coating, not the coating itself. Moreover, regardless of the type of primers, they did not significantly affect the heating temperature of the elements or the clarity of the image.

**Figure 7:**
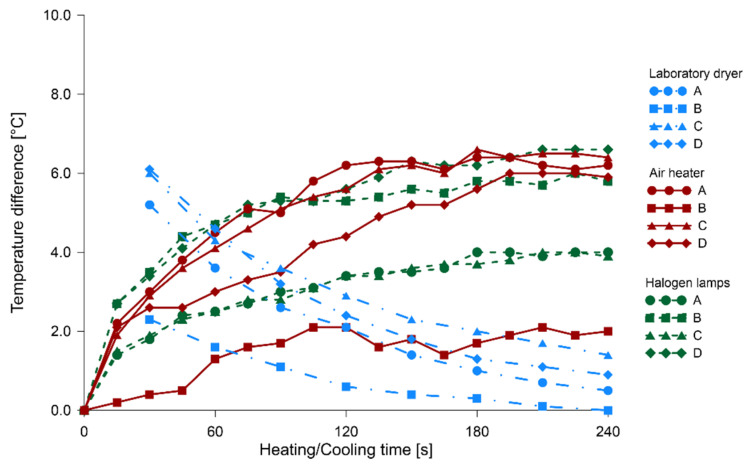
The relationship between heating/cooling time and temperature difference of solid fir wood and knots.

**Figure 8:**
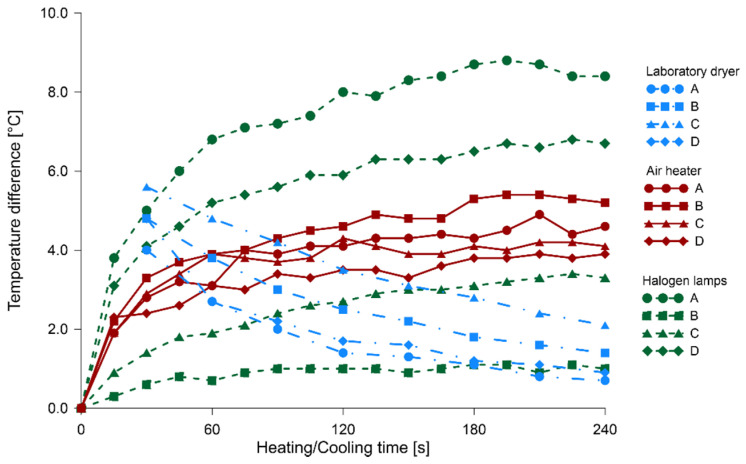
The relationship between heating/cooling time and temperature difference of solid pine wood and knots.

**Figure 9:**
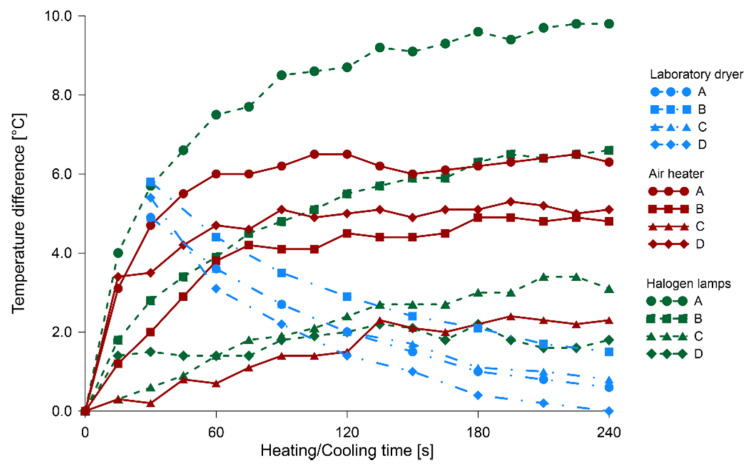
The relationship between heating/cooling time and temperature difference of solid spruce wood and knots.

In Figure 7, Figure 8 and Figure 9, the relationships between heating/cooling time and temperature difference of solid wood and knots are illustrated, respectively, for fir, pine and spruce specimens. The blue color indicated the process of timber cooling after its heating in the laboratory dryer; the thermal images started to be taken after 30 s of leaving the dryer. Heating processes with the use of air heater and halogen lamps were illustrated, using respectively red and green color. The respective times for which temperature differences were determined were connected with lines (different type for each method) for the visual representation of the process.

The cooling of knots after heating them in a laboratory dryer is approximately exponential, thus leading to temperature stabilization, as well as equalization. Therefore, it can be concluded that knots tend to cool down more slowly than solid wood. An analysis of the temperature change curves during the heating of the sample with both halogen lamps and the air heater indicated that the temperature difference between the control point located on the knot and one on the solid wood was increasing to a value of about 1.5 min after the beginning of the heating process, and then the difference levelled out and kept approximately constant. Determining the minimum heating time to obtain satisfactory results is important, given the deteriorating influence of light and elevated temperatures on polychromes.

### 3.2. Knot Area Ratio

**Figure 10:**
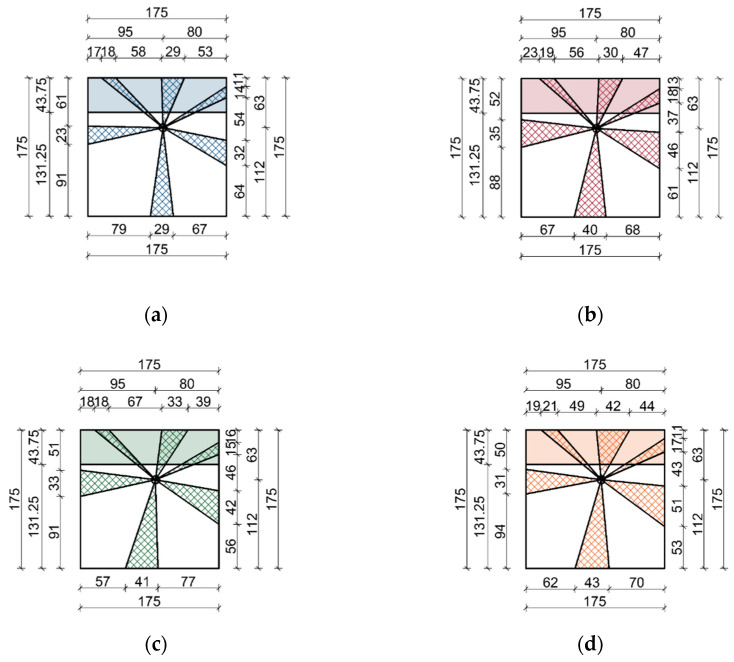
Knot area ratio in the critical cross-sections of a pine sample in the test: (**a**) macroscopic, (**b**) laboratory dryer, (**c**) air heater and (**d**) halogen lamps.

For purposes of the analysis, the dimensions of the elements were averaged, and the sides of the samples were assumed to be 175 mm long. In reality, the lengths of the sides of the samples ranged between 173 and 178 mm. After preliminary examination, it was established that such approximation would not affect the results significantly. To determine the knot area ratio, an auxiliary critical cross-sectional pattern was made for both geometrically measured knots and those measured from scaled thermal images. For the determination of KAR using thermal imaging, a critical cross-sectional pattern was identified for the areas with the highest proportion of knots based on thermal images taken on each surface. The determined planes of projection are shown in Figure 10 for the pine sample. Corresponding sets of illustrations were prepared for the remaining samples. Subsequently, on their basis, the specimen cross-sectional area, the margin area and the proportion of knots in these cross-sections were calculated.

Table 2 presents the results of the total and the margin knot area ratio, including the worse margin for each of the test phases: macroscopic tests, and three thermographic tests with a different energy source. On this basis, the elements were classified into timber grading strength classes (KW—best quality, KS—medium quality and KG—inferior quality), as per visual sorting according to Reference [44]. Moreover, taking into account the wood species and grading strength class, the strength class of elements was matched according to the standard [51].

In the assessment of the KAR, the size and position of the knot in relation to the pith of the section are crucial. It is important to note the significant influence of the external energy supply in active thermographic examination and the error associated with the selection of the temperature range of thermal images (in this case, between 25 and 43 °C) and the visual assessment of the images by the researcher on the interference of the KAR calculation. The chosen images to determine the TKAR and MKAR were selected for each heating method individually: For the laboratory dryer, it was the first image taken after 30 s from the start of the cooling process; for the air heater, the images were taken after 2–3 min of heating, and in the case of halogen lamps, the image were taken after 3–4 min. The mean values of TKAR and MKAR for the fir sample were, respectively, 0.13 and 0.12, for the pine sample 0.2 and 0.21 and for the spruce sample 0.16 and 0.32.

The knot area ratios (TKAR and MKAR) macroscopically defined for elements made of pine and fir timber allow us to classify them to the grading strength class, KW, which is the highest grading class and the spruce element to the lower type of class—KS—due to their higher proportion of knots in the margin zone.

For the fir element, the values of KAR determined macroscopically were relatively low, which made the element remain in the same grading strength class at each stage of testing, despite the increase of their values in the thermal imaging estimation. On the contrary, in the case of the pine element, which had initially a higher KAR (close to the limit of KW and KS classes), in the case of heating with halogen lamps, a significant increase in its value caused the element to be classified in the worst class, namely KG class. It is related to complex polychrome and unfavourable localization of knots. What is interesting is that the grading strength class of spruce wood remained unchanged throughout the entire scope of tests. However, the increase in the value of KAR was comparable to that of the other specimens. In the case of external energy source in the form of an air heater, grading strength class remained the same, which may suggest that this technique can be used for different types of paint coatings and wood species in real elements. It is subject to the slightest error associated with different paint characteristics.

### 3.3. Semi-Destructive Test

**Figure 11:**
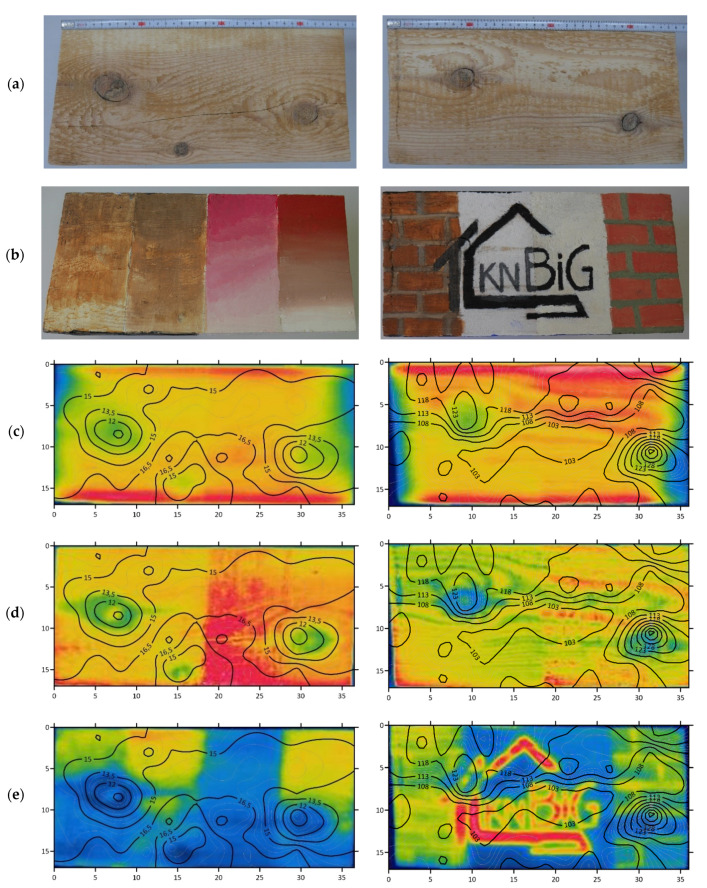
A comparison of the semi-destructive tests (SDTs) and thermographic tests (left—surface B and right—surface D): (**a**) surfaces before painting, (**b**) surfaces with paint coatings, (**c**) SDT results compared to thermal images of surfaces after preheating in laboratory dryer, (**d**) SDT results compared to thermal images during air heating and (**e**) SDT results compared to thermal during heating with halogen lamps.

The results of SDTs from individual points underwent estimation with the ordinary kriging method, which allowed us to obtain the best, unbiased linear estimator. In the ordinary kriging method, the local means are not closely related to the average from the whole sample population, and during the average estimation, only points from the local neighbourhood are selected, thus creating an isoline of the distribution of results of drilling resistance tests—RM—and penetration depth of the sclerometer needle—PD. It should be remembered that the results at the boundary of the estimation area may be distorted. Figure 11 shows the analyzed surfaces: digital images before and after the application of paint coatings and the integration of thermal images with the distribution isolines of the SDTs obtained by the kriging method.

On the analyzed surfaces, sound, partially grown-up knots were found; also, the wood grain for the tangential section was clearly visible. There was also a crack on surface B (Figure 11a). For both sclerometric and resistographic tests, strong correlations with density are confirmed: The higher the density, the lower PD, and the higher the RM [10,52,53]. The average PD for the whole sample surface was 15.12 mm, while the average RM was 111.23. The distribution of sclerometric results (surface B) clearly indicated an increase in wood density within two larger knots (PD ≈ 12 mm); within the area of a small knot, a decrease in penetration depth determined by the kriging method is not significant (PD ≈ 15 mm). The results of the drilling resistance test (surface D) correctly reflect the distribution of knots (RM > 123).

Both for sclerometer and drilling resistance tests, the isolines generated by the kriging method do well when recreating the temperature distributions obtained for tests on the specimen after preheating in laboratory dryer and during air heating. On the other hand, the comparison of thermal images taken during heating with halogen lamps with the SDT obtained isolines indicates considerable discrepancies, which is connected with this method of heating. Correct representation of the distribution of knots by both the thermal imaging method after preheating sample in laboratory dryer and during air heating, combined with SDT methods, is associated with their increased density, which, in the case of active thermographic tests, results in slower heating, and, in the case of SDT tests, an increase in drilling resistance and a decrease in penetration depth. It should be considered that the analyzed knots were sound and at least partially grown-up—for decayed or falling out knots, these relationships might not be that clearly defined.

## 4. Discussion

The generated thermal images are strongly influenced by the source of external energy supply. It should be considered that the tests were carried out with a single emissivity, which could cause a disturbance in the temperature registration by the IR camera—an overestimation or underestimation of temperature in comparison to reality. In particular, this applies to the heating with halogen lamps where there was a noticeable error related to the reflected radiation. On the other hand, the temperature difference between the surface of knots and solid wood was analyzed, not the temperature values themselves. The use of a fixed value of the emissivity (ε = 0.95) was an intentional procedure, intended to reproduce possible in situ tests conditions. In the research of historical buildings, it is rare to deal with material with a constant and known emissivity.

**Figure 12:**
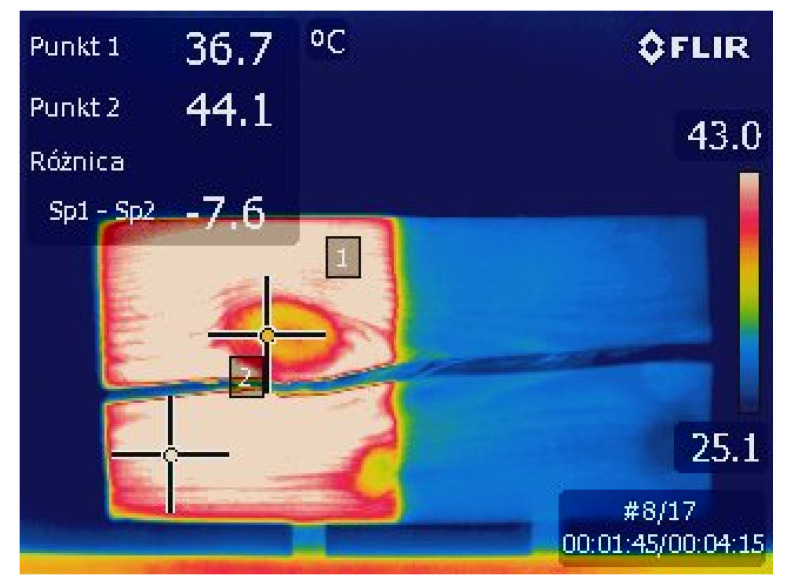
An example of a thermal image of a pine sample (surface A) during continuous heating with halogen lamps.

In the case of tests on preheated samples in a laboratory dryer, the obtained thermal images showed almost no difference in the type of paint coating, but a wood grain was clearly visible. The defects of the wood itself, such as knots, were relatively noticeable, but the border between the knot and the surrounding solid wood was not too sharp. For thermal images obtained during surface heating with an air heater, the boundaries between different types of paint layers became more distinct, but no ornaments were evident. The wood grain was not as sharp as in the case of free convection. However, the knot borders became sharper and clearer. The use of halogen lamps in active thermography of wood provided a strong optical indication of the polychrome. Nevertheless, the wood grain and the occurrence of defects were not as obvious in perception as in previous methods. It is connected with a significantly increased phenomenon of reflection, which occurs when halogens lamp is used. In addition, the colors of the paint had a substantial impact on the thermal images. In the case of surfaces covered with a homogeneous paint color, the thermal radiation method provides a relatively accurate estimation of knot size (Figure 12). In the literature, there is a practice of painting low-emissive surfaces with high-emittance flat black paint [54]; however, in the case of historical elements, in which polychromes are often cultural heritage, such a practice is not possible for preservation concerns.

The most unified course of results was found in the readings of the change in temperature difference during the cooling of specimens. After 30 s from the end of the heating process, the average temperature difference between a knot and solid wood was 5.0 °C, with a standard deviation of 0.8 °C. In the case of 90 s heating with the air heater, these values were 4.0 and 1.2 °C, respectively, and for halogen lamps 4.1 and 2.1 °C. The highest scatter of values was observed for halogen lamps due to impaired registration of temperature measurements. Partially similar tests were performed on a sample of Pinus pinaster with three induced holes involving halogen heaters [32]. In this case, the wood sample surface was black painted, to guarantee the emissivity value fixed in 0.95. Comparing the graphs of temperature changes during heating and cooling in our research and those of the above mentioned studies, it can be observed that the courses of these variations are similar in shape, in spite of using a different source of external energy.

Regardless of the external energy supply method, the size of knots influenced the results of thermal imaging tests. The smaller the knot, the faster it heats up and is generally less detectable by the device. The smallest knots (e.g., knot on surface B in a fir sample) only heat up 2 °C less than the surrounding solid wood. Moreover, the type of knot determines the quality of the measurement. The temperature in decayed or cracked knots is distributed differently than in sound knots.

The analysis of the KAR for each stage of the investigation showed relatively encouraging results in terms of the potential use of active thermal imaging for this purpose. However, it should be noted that the values of KAR based on thermal images are overestimated in relation to the actual values for each of the examined wood species. The reason may be the anatomical structure of wood surrounded by defects or the accuracy of the measurement method. The knots interfere with the homogeneity of the wood structure both as a result of the different fiber direction and by displacing the fibers of the surrounding wood from the parallel direction. In the region of knots in coniferous wood, there is also a possibility of occurrence of reactive wood with increased density in comparison to sound wood. Therefore, the abnormalities in the thermal imaging may be more extensive than the actual knot size, which is also confirmed by semi-destructive tests.

Analyzing the alterations in the total KAR values obtained on the basis of thermographic images, as compared to macroscopic examinations, in the case of the laboratory dryer, the average difference for all wood species was 0.07. A similar value of the average difference (0.06) was obtained for heating with halogen lamps. The lowest average difference between macroscopic examination and results from thermal images of 0.04 was found for air heater. Moderately comparable results were derived for the comparison of the margin KAR. Nevertheless, an overestimation of the KAR values may not lead to a decrease in the grading strength class, due to their wide scale.

Recognizing the difficulties in application and the errors generated, the authors maintain that active thermography is applicable in the diagnostics of historical wood covered with polychromes. In the literature, there are studies of experiments using both passive and active infrared thermography to detect internal defects of wood [28]. Using two methods of heating with a laboratory dryer and halogen lamps, the researchers drew similar conclusions and noted the high potential of active thermography as non-destructive testing of the internal structure of wood, regardless of its moisture content. These tests were performed on artificially prepared defects.

In this work, subjective visual evaluation of thermal images was applied; however, it is also possible to incorporate automatic computing algorithms. For example, in the patent US 7,778,458 B2, a method for searching knots in the wood, wherein a piece of wood is photographed by photographing means, degrees of circularity are calculated from the photographed images of the piece of wood, and an image of figure with a significant degree of circularity is deemed a knot candidate, was introduced [55]. Automatic evaluation of knots would increase accuracy, as well as eliminate human error. Moreover, another future possibility is the use of the automatic device [56] for the examination of objects with the active thermography method would allow for the application of the elaborated procedures in the inspection of historical timber elements, in particular ceilings. Depending on the type of ceiling surface finish, it would be possible to approximate the KAR and to precisely indicate the locations for semi-destructive tests.

Under in situ conditions, active thermography will require the use of working platforms that allow the operator with equipment to approach the examined element at a sufficient distance. It should also be noted that, in real testing conditions, there may not always be a possibility of performing measurements with the camera and energy source oriented perpendicular to the specimen. This setup would complicate yet not necessarily preclude the determination of KAR or the identification of a semi-destructive test location. In this case, if the camera is not positioned perpendicularly to the specimen, the imaging of the surface structure may be performed by using a series of photographs, in accordance with algorithms known from photogrammetry (measurements from photographs taken at a known angle). On the other hand, if the energy source cannot be set at a right angle, one may attempt to eliminate the problem with two sources oriented angularly at a given object (as performed in the halogen lamps setup). However, in the case of one-sided measurements, it is necessary to take into consideration the uneven heating of the object.

## 5. Conclusions

The paper covered the topic of application of active thermography in determining the knot area ratio of elements covered with paint coatings that simulated historic polychromes. The preservation of the polychrome contributes to the maintenance of the wooden Renaissance cultural heritage. The performed tests and analyses provide the following statements:

- Defects of timber are exposed in the thermal images due to the decreased temperature in relation to solid wood. In the whole scope of research, for thermal images taken during the sample heating after 90 s, the difference between knots and solid wood was approximately 4 °C; for images taken during the sample cooling after 30 s, the difference between knots and solid wood was approximately 5 °C, which allowed us to determine the approximate position of knots within the element.
- The contribution of knots in the piece of wood is one of the basic features determining its strength. Therefore, the possibility of determining the knot area ratio in polychromed historical buildings is an important and ongoing concern. The conducted research indicated that it is possible to estimate the knot area ratio by using active thermography; however, it is likely to be affected by an error. The limitation of this determination method is the necessity to access the element from four sides and the relatively subjective measurement of the knot diameter on the thermal image.
- The comparisons of semi-destructive and thermographic tests showed a great potential of using active thermography to determine the zones for NDT and SDT tests of polychromed timber elements. The use of active thermography prior to performing such tests prevents misinterpretations associated with unintentional measurements in the area of natural defects of wood (such as knots) and may also reduce the number of measurement areas necessary to properly assess the technical condition and thus the extent of damage caused by the examinations.
- The paper describes three methods of energy supply to the samples and their influence on the values of the estimation of the KAR and the possibility of determining the locations for further SDTs. In terms of practical applications in historic buildings, halogen lamps would be the most convenient; however, the results of those (KAR values and correlation with the SDTs) were rather insufficient. The supply of heat through convection would be more troublesome due to the availability of equipment and power source. In principle, without dismantling the element, it is rather not possible to use free convection (laboratory dryer), for which the most obvious wood grain and defect location is obtained regardless of the type of paint coating. Therefore, it seems that, in terms of conservation practice, the most suitable solution would be to use an air heater, for which, despite the influence of the type of paint coating on the thermal image, it was possible to determine the location of possible defects or the value of knot area ratio.
- The further research in the field of active thermography for the detection of natural defects in polychrome decorated timber should be focused on the application of post-processing methods such as Eulerian video magnification for air heating [57,58] and quantitative evaluation, using noise-related parameters. However, using energy sources that are hazardous to polychromes should be avoided, especially devices that emit excessively high UV radiation (e.g., Xenon flash lamps), sources generating elevated temperatures (higher than 45 °C) and techniques that require direct contact between the source and the examined object. It would also be beneficial to develop an algorithm for determining the KAR and proper locations for semi-destructive testing in the case of elements which could not be tested perpendicularly.

## Figures and Tables

**Table 1:** Primers and paints coating corresponding to the surface area used in the experimental programme.

Surface	A	B	C	D
Primer	None	Construction Putty	Glue–Chalk Plaster	None
Paint coating	Alkyd paint		Protein binder with pigment	
			Klucel with pigment	
			Acrylic paint	
			Oil paint	

**Table 2:** Knot area ratio in the critical cross-section of the elements with classification to quality and strength class, according to References [44,51], in the whole scope of research.

Wood Species	Type of Test	Total KAR	Margin KAR	Grading Strength Class	Strength Class
Fir	Macroscopic	0.11	0.12	KW	C22
	Laboratory dryer	0.19	0.18	KW	C22
	Air heater	0.16	0.13	KW	C22
	Halogen lamps	0.17	0.15	KW	C22
Pine	Macroscopic	0.19	0.23	KW	C35
	Laboratory dryer	0.26	0.25	KS	C24
	Air heater	0.25	0.25	KW	C35
	Halogen lamps	0.28	0.31	KG	C20
Spruce	Macroscopic	0.17	0.35	KS	C24
	Laboratory dryer	0.25	0.48	KS	C24
	Air heater	0.19	0.41	KS	C24
	Halogen lamps	0.21	0.38	KS	C24

## Data Availability

The data that support the findings of this study are available from the corresponding author upon request.
